# Whole-genome sequencing of *Lactobacillus helveticus* HP-B1137 and its biotransformation capacity toward calycosin-7-O-β-D-glucoside

**DOI:** 10.3389/fmicb.2026.1962579

**Published:** 2026-09-30

**Authors:** Shu-Hang Zhang, Lan-Fang Li, Zhao-Sen Fan, Shao-Yang Hou

**Affiliations:** 1 College of Pharmacy, Heze University, Heze, China; 2 Shandong Benon Biological Technology Co., Ltd., Heze, China

**Keywords:** biotransformation, calycosin, complete genome, gene annotation, *Lactobacillus helveticus*

## Abstract

**Introduction:**

*Astragalus*, as a traditional tonic herb, has alycosin-7-O-β-D-glucoside as one of its main active components.

**Methods:**

In this study, a strain of *Lactobacillus helveticus* HP-B1137 was isolated from raw milk in Mudan District, Heze City, Shandong Province. The probiotic properties, and ability to biotransform calycosin-7-O-β-D-glucoside were systematically evaluated.

**Results and discussion:**

Genome analysis revealed that HP-B1137 possesses a circular chromosome (2,098,901 bp) comprising 2,161 CDs, 63 tRNAs and 12 rRNAs. Two circular plasmids were also identified, with sizes of 23,906 bp and 6,414 bp and GC contents of 38.54 and 34.49%, respectively. Functional annotation showed that the strain is rich in carbohydrate metabolism genes, with 29 glycoside hydrolase genes annotated by the CAZy database, providing a genetic basis for its glycoside-hydrolyzing capability. *In vitro* probiotic tests demonstrated that the strain maintained high viable cell counts after 3 min of heat treatment at 50 °C, achieved a survival rate of 108.33% after 150 min in artificial gastric juice at pH 2.7. It also inhibited 11 pathogenic bacteria, producing inhibition zones ranging from 9.83 to 16.37 mm. Whole-cell transformation experiments indicated that HP-B1137 could nearly completely convert 0.5 mg/mL calycosin-7-O-β-D-glucoside into calycosin within 36 h. Further cloning and expression of the recombinant BgsH enzyme revealed optimal reaction conditions at 37 °C and pH 7.0; under optimized conditions—0.1% substrate concentration, 10 U/mL enzyme activity, and 12-h incubation—the conversion efficiency exceeded 96.3%. In summary, *L. helveticus* HP-B1137 is a functional strain integrating efficient biotransformation, excellent probiotic characteristics, tolerance to extreme environments, and broad-spectrum antimicrobial activity, showing significant potential for developing functional foods derived from *Astragalus* and green bio-manufacturing of active aglycones.

## 1 Introduction

*Astragalus* as a traditional Chinese medicine for strengthening the qi (Zhang et al., 2024), has the functions of strengthening qi, raising yang, consolidating the exterior and stopping sweating, as well as promoting diuresis and reducing swelling. The saponins and flavonoids it contains are its main active components (Chen et al., 2020). Calycosin-7-O-β-D-glucoside (CAG) and calycosin (CA) are two of the main isoflavone compounds found in astragalus. CAG is one of the most active main components of astragalus (Deng et al., 2021), has obvious effects such as antioxidant, antiviral, inhibition of melanin formation and immune regulation (Liang et al., 2026; Wang et al., 2026). However, the activity of CA is more prominent-this compound has estrogen-like effects and has shown excellent results in anti-inflammatory, anti-tumor, antioxidant, and anti-viral aspects. Its anti-inflammatory activity has promising application prospects in protecting and improving inflammatory damage to various organs (Comlekcioglu, 2024; Han et al., 2022). On the other hand, CAG has larger molecules and is difficult to be absorbed directly through intestinal cell membranes, resulting in a relatively low bioavailability, In contrast, CA has smaller molecules and better lipid solubility, enabling it to more efficiently penetrate cell membranes and thus achieving a higher bioavailability (Deng et al., 2021). In natural astragalus, the content of CA is relatively low and its extraction is rather difficult, which limits its large-scale application (Liang et al., 2026). Therefore, in pharmaceutical and food applications, it is necessary to hydrolyze CAG to convert the sugar groups into CA components, in order to increase the absorption rate in the human body and enhance the pharmacological activity. The traditional conversion method employs acid hydrolysis (Liang et al., 2026; Pan et al., 2021; Shao et al., 2011). However, this method has problems such as low conversion rate, generation of harmful impurities, and severe environmental pollution. Compared with the traditional chemical hydrolysis method, the use of β-glucosidase BgsH (Lee et al., 2025) to convert glucose derivatives into aglycones (Otieno and Shah, 2007), significant advantages such as high specificity of the reaction, high conversion efficiency, and environmental friendliness have been achieved. This has made it a research hotspot in recent years. In nature, microorganisms capable of producing BgsH are widely distributed, including *Bacillus subtilis,* (Inagaki, 2025; Kuo and Lee, 2008), *Lactobacillus plantarum* (Marasco et al., 1998), and *Paenibacillus polymyxa* (Isorna et al., 2007) among others. However, when using these traditional production hosts for industrial production of β-glucosidase or directly applying it in food fermentation, there are generally the following limitations: low enzyme production levels, inhibition regulation by carbon decomposition metabolites, and the lack of probiotic functions in the strains themselves (Khadye et al., 2021; Tavares et al., 2023), making it difficult to balance efficient conversion and health benefits. Therefore, the screening of food-grade microorganisms and the optimization of enzyme production conditions remain important research directions in this field.

*L. helveticus* is a type of thermophilic, homofermentative, Gram-positive lactic acid bacteria (Chelladhurai et al., 2023), belonging to the *Lactobacillaceae* (Zhao et al., 2011). It was first described by Orla-Jensen in 1919 and was officially named in 1925 (Naser et al., 2006). This strain is one of the most protein-degrading members in the lactic acid bacteria family (Griffiths and Tellez, 2013), and is widely found in cheese, fermented milk, and other dairy products (Toropov et al., 2018), and consequently plays a significant role in the dairy industry. The *L. helveticus* has unique metabolic characteristics: it can metabolize galactose (Liu C. et al., 2025), and has a high acid production rate (Lee et al., 2026), strong proteolytic activity (Griffiths and Tellez, 2013), and can produce various bioactive peptides. These characteristics enable it to not only improve the flavor, texture and aroma of the final product during the fermentation process, but also significantly enhance the nutritional value and health benefits of the product (Chelladhurai et al., 2023; Li et al., 2025). Based on its long history of safe use, *L. helveticus* has been included in China’s “List of Bacterial Strains Suitable for Food Use,” and is recognized as a safe food-grade microorganism (Du et al., 2024). This study found that a strain of *L. helveticus*, HP-B1137, can completely convert CAG into CA, and at the same time has extremely strong gastrointestinal tolerance, broad-spectrum antibacterial activity and genomic-level safety, enabling an integrated application of “fermentation, conversion, and gain” and having significant advantages in the development of functional foods and health supplements using astragalus as the raw material.

The rapid development of whole-genome sequencing technology has provided powerful tools for the safety assessment of probiotics and the exploration of functional genes (Jiang et al., 2023; Peng et al., 2023). Through whole-genome sequencing and functional annotation of the strains, not only can the potential virulence factors and antibiotic resistance genes of the strains be comprehensively evaluated at the molecular level to eliminate safety risks (Liu et al., 2026), but also functional gene clusters related to probiotic properties, such as glycoside hydrolase genes, adhesin genes, and bacteriocin biosynthesis genes, can be systematically identified (Stergiou et al., 2021). However, current research on *L. helveticus* in the biotransformation of flavonoid glycosides is still relatively limited. Most existing studies focus on other *Lactobacillus* species such as *L. plantarum* (Guo et al., 2020; Lee et al., 2022), while whether *L. helveticus* has the ability to efficiently convert CAG and its conversion efficiency are still lacking systematic reports. More importantly, *L. helveticus* strains with excellent probiotic properties, broad-spectrum antibacterial activity, genome-level safety, and efficient CAG biotransformation ability are even scarcer. Based on this, this study isolated a *L. helveticus* HP-B1137 from traditional fermentation samples and conducted whole-genome sequencing and annotation. Combined with whole-genome sequencing and multiple database functional annotation, including COG, KEGG, GO, Pfam, CAZy, CARD/ARDB, and VFDB, systematic *in vitro* probiotic property evaluation and HPLC dynamic monitoring of CAG biotransformation were carried out to comprehensively evaluate the probiotic potential, safety, and biotransformation efficiency of this strain, aiming to provide high-quality strain resources and scientific basis for the development of functional foods and the green biological manufacturing of active ingredients from traditional Chinese medicine. At the same time, the enzymatic properties of this strain were purified and characterized, the key enzyme for CAG conversion to CA was identified, and its application potential in CA industrial production was further explored.

## 2 Materials and methods

### 2.1 Strain culture conditions and plasmids

The strain used in this study was isolated from raw milk in Mudan District, Heze City, Shandong Province, and was identified by 16S rRNA gene sequence and then stored in our laboratory. *Escherichia coli* DH5α and BL21 (DE3) competent cells were used for plasmid cloning and protein expression. The plasmid pET-28a (+) (Novagen) was used as the expression vector for the *BgsH* gene, which carried a His-Tag label and a kanamycin resistance gene and was suitable for the purification of C-terminal fusion proteins in the prokaryotic expression system. MRS medium was purchased from Beijing Luqiao, and LB medium (10 g/L peptone, 5 g/L yeast extract, 10 g/L NaCl) was used for *E. coli* cultivation. *L. helveticus* HP-B1137 was cultured statically at 37 °C under anaerobic conditions in MRS solid or liquid media; *E. coli* was cultured at 37 °C, 200 r/min in an orbital shaker, and the heterologous expression plasmid required 50 μg/mL kanamycin.

### 2.2 Isolation, purification, and phylogenetic analysis of *Lactobacillus helveticus* HP-B1137

The collected fermentation samples were diluted to concentrations of 10^−5^, 10^−6^, and 10^−7^ using sterile physiological saline. 100 μL of the diluted solution was spread onto MRS solid plates and cultured under anaerobic conditions at 37 °C for 48 h. Single colonies with typical characteristics were picked and repeatedly passaged and purified three times through continuous three-zone streaking until pure cultures were obtained. The purified strains were stored in MRS liquid medium containing 20% glycerol at −80 °C for long-term preservation. The genomic DNA of *L. helveticus* HP-B1137 grown in the logarithmic phase was extracted using the HiPurA™ bacterial genomic DNA purification kit (Thermo Fisher Scientific, USA) (Ganesh et al., 2025). Through nanopore sequencing and Illumina short-read sequencing technologies, the complete genome sequence of HP-B1137 was obtained. The extracted genomic DNA was used as a template for PCR amplification using bacterial 16S rRNA universal primers. The obtained 16S rRNA gene sequences were subjected to BLAST comparison in the NCBI database, multi-sequence alignment was performed using the CLUSTAL X program, and a phylogenetic tree was constructed using the neighbor-joining method in the MEGA software package (version 7.0.26) for molecular identification and phylogenetic analysis.

### 2.3 Genome annotation and analysis

For the assembled genomic sequences, the protein-coding genes (CDS) were predicted using the Prodigal software (version 2.6.3); tRNA and rRNA genes were predicted using tRNAscan-SE (version 2.0) and Barrnap (version 0.9) respectively. The predicted gene sequences were compared to the following public databases for functional annotation. (1) The COG^1^ database was used for direct homology classification using BLASTP (E-value≤1e^−5^); (2) The KEGG^2^ database was used to annotate the metabolic pathways in which the genes were involved; (3) The GO^3^ database was used to annotate the gene functions from three dimensions: cellular components, molecular functions, and biological processes; (4) The Pfam database was annotated using the HMMER software (version 3.3.2) for protein domain family annotation; (5) The UniProt, RefSeq, and NR databases were used for comprehensive protein function and homology annotation. Carbohydrate-active enzymes were annotated using the Carbohydrate-Active Enzymes (CAZy) database, with a focus on glycoside hydrolase (GHs) family genes. Resistance genes were screened using Comprehensive Antibiotic Resistance Database (CARD) and Antibiotic Resistance Genes Database (ARDB); virulence factors were annotated using Virulence Factors Database (VFDB). Signal peptide prediction was performed using SignalP 5.0, and transmembrane helix prediction was performed using TMHMM v2.0.

### 2.4 Thermotolerance assay of *Lactobacillus helveticus* HP-B1137

Using bamboo sticks, the single colonies of *L. helveticus* HP-B1137 on the MRS solid medium were inoculated into the freshly sterilized MRS liquid medium. The culture was conducted under anaerobic conditions at 37 °C, with a speed of 180 r/min for 18–24 h. The bacterial solutions were heated for 1 min, 2 min, and 3 min at 37 °C, 50 °C, and 70 °C, respectively. After dilution to a certain concentration, the bacterial solutions were spread onto the solid MRS medium and incubated anaerobically at 37 °C for 18–24 h. The single colonies on the plates were counted.

### 2.5 Determination of acid production and acid tolerance of *Lactobacillus helveticus* HP-B1137

Using bamboo sticks, the single colonies of *L. helveticus* HP-B1137 on the MRS solid medium were inoculated into the freshly sterilized MRS liquid medium. The culture was conducted under anaerobic conditions at 37 °C and 180 r/min. After culturing for 12 h, 21 h, and 26 h respectively, centrifuge for 15 min, the pH of the supernatant was measured.

Prepare artificial gastric juice (8.2 mL of dilute hydrochloric acid, 400 mL of double-distilled water, 5 g of pepsin, mix well and add double-distilled water to make a total volume of 500 mL. Take a certain amount of the solution and adjust the pH to 2.7, 3.46, and 4.75. Sterilize with a 0.44 μm filter membrane, store at 4 °C in the refrigerator for future use). Use a bamboo stick to inoculate the single colony of *L. helveticus* HP-B1137 on the MRS solid medium into the freshly sterilized MRS liquid medium. Cultivate at 37 °C, 180 rpm in an anaerobic environment for 18–24 h. Take 60 mL of the bacterial solution, centrifuge at 3580 rpm for 15 min, discard the supernatant, resuspend the bacteria with 1.5 mL of physiological saline. Take 3 centrifuge tubes, respectively add 6 mL of artificial gastric juice, then add 200 μL of the suspended bacterial solution and mix well. Dilute to a certain concentration at 0 min and 150 min and spread the plates onto the solid MRS medium for 18–24 h of cultivation. Then count the single colonies on the plates and calculate the survival rate.

### 2.6 Determination of bile salt tolerance of *Lactobacillus helveticus* HP-B1137

Prepare artificial intestinal fluid, 3.4 g disodium hydrogen phosphate, 250 mL double-distilled water dissolved, adjust pH to 6.8 with 0.1 mol/L sodium hydroxide solution, add 2.5 g trypsin, shake well, then add double-distilled water to make a total volume of 500 mL. Take a certain amount of the solution and add a certain amount of bovine bile salts to prepare artificial intestinal fluids with concentrations of 0.03, 0.1, 0.2, and 0.3%. Filter sterilize with a 0.44 μm filter membrane, store at 4 °C in the refrigerator for later use. Use a bamboo stick to inoculate the single colony of *L. helveticus* HP-B1137 on the MRS solid medium into the freshly sterilized MRS liquid medium. Cultivate anaerobically at 37 °C, 180 rpm for 18–24 h. Take 60 mL of the bacterial solution, centrifuge for 15 min, discard the supernatant, resuspend the bacteria with 1.5 mL physiological saline. Take 4 centrifuge tubes, respectively add 6 mL of artificial intestinal fluid, then add 200 μL of the suspended bacterial solution and mix well. Dilute to a certain concentration and spread the plates onto the solid MRS medium for 18–24 h of cultivation. Then count the single colonies on the plates and calculate the survival rate.

### 2.7 Determination of antibacterial activity of *Lactobacillus helveticus* HP-B1137 fermentation broth

The antibacterial activity of *L. helveticus* HP-B1137 fermentation broth against indicator bacteria was determined using the Oxford cup agar diffusion method. Using a bamboo stick, the single colonies of *L. helveticus* HP-B1137 on the MRS solid medium were inoculated into fresh sterilized MRS liquid medium. The culture was conducted at 37 °C, 180 r/min in an anaerobic environment for 18–24 h. A certain amount of the bacterial solution was taken, centrifuged at 8000 r/min for 1 min, and the supernatant was collected. Another certain amount of the bacterial solution was reserved. The mixed bacteria were inoculated onto LB solid medium, and then transferred to LB solid medium. After the mixed bacterial plates dried slightly, four Oxford cups with a diameter of 8.0 mm were placed evenly on them, and left to stand for 5 min. Two Oxford cups were added with 200 μL of the supernatant, and the other two Oxford cups were added with 200 μL of the undistilled bacterial solution. They were placed in a 4 °C refrigerator and incubated upright at 4 °C for 4 h, and then placed at 37 °C for upright culture for 24 h. The inhibition diameters were observed and recorded. As a negative control, MRS broth adjusted to the same pH as the fermented supernatant was used to exclude the inhibitory effect caused by acidification.

### 2.8 Cloning and heterologous expression of the *β-D-glucosidase* (*BgsH*) gene of *Lactobacillus helveticus* HP-B1137

Based on the gene sequence annotated as *6-phospho-β-D-glucosidase* (*BgsH*) in the complete genome sequencing result of *L. helveticus* HP-B1137, specific primers were designed. Forward primer: 5′-atgacatttccaaagaactttttgt-3′, reverse primer: 5′-ttaacctaaatcttcaccatttgaagc −3′. PCR amplification was performed using the genomic DNA of HP-B1137 as the template. The amplified BgsH gene fragment was constructed into the pET28a (+)-BgsH expression plasmid using the Red/ET cloning technology and the GB05-dir recombinase, and transformed into the *Escherichia coli* BL21 (DE3) strain for heterologous expression. Positive clones were screened on LB plates containing 50 μg/mL kanamycin. The cloned gene was amplified using universal primers for T7/T7-term amplification to confirm the completion of the target vector construction. The correctly sequenced *Escherichia coli* BL21 (DE3) was spread on kanamycin-containing LB plates and cultured at 37 °C overnight. A single colony was picked and inoculated into LB liquid medium containing kanamycin, and cultured at 37 °C, 200 r/min, for 20 h until OD₆₀₀ was approximately 0.6–0.8. IPTG was added to a final concentration of 0.1–1.0 mmol/L to induce expression at 16–37 °C for 4–16 h. The uninduced recombinant bacteria and the bacteria containing an empty vector were used as controls. After induction, the bacteria were centrifuged at 4 °C, 8,000 r/min for 10 min, and the supernatant (soluble protein) and precipitate (inclusion body) were collected. The supernatant and precipitate were resuspended in binding buffer (20 mmol/L Tris–HCl, 500 mmol/L NaCl, 20 mmol/L imidazole, pH 8.0) and subjected to ultrasonic disruption (power 300 W, 3 s on, 5 s off, total time 15 min) followed by centrifugation at 12,000 r/min for 20 min. The supernatant (soluble protein) and precipitate were collected separately for SDS-PAGE analysis to determine the expression form and quantity of the recombinant BgsH protein. Then, Ni affinity chromatography column was used with a pH 7.4 elution buffer (containing 300 mmol/L NaCl, 50 mmol/L NaH₂PO₄, 50–200 mmol/L imidazole and 10 mmol/L Tris base) for gradient elution to separate the target protein for SDS-PAGE analysis. The protein concentration was determined using the Bradford method with bovine serum albumin as the standard.

### 2.9 Biotransformation of calycosin-β-D-glucoside into calycosin catalyzed by recombinant BgsH

Whole-cell transformation: A single colony of *L. helveticus* HP-B1137 was inoculated into 30 mL of freshly sterilized MRS liquid medium and cultured anaerobically at 37 °C, 180 r/min for more than 6 h. Then, CAG standard was added to the final concentration of 0.5 mg/mL and the culture was continued to be incubated anaerobically at 37 °C, 180 r/min for 24 h. Samples were taken at 0, 12 and 24 h, and an appropriate amount of fermentation broth and extraction agent (ethyl acetate) were added, shaken and mixed. The bacterial suspension was centrifuged at 3580 r/min for 10 min, and the topmost clear liquid was collected. It was then subjected to rotary evaporation at 55 °C, dissolved in 10% acetonitrile, and analyzed by high-performance liquid chromatography according to the reference method, with some modifications (Tan, 2026). The conversion efficiency was calculated as ([CAG]₀ − [CAG]ₜ) / [CAG]₀ × 100%, based on the integrated peak areas from HPLC and standard curves for CAG and CA.”

Biological transformation of purified enzyme solution: Take the above purified BgsH enzyme solution (10 U/mL) and CAG (0.5 mg/mL) and react them at an appropriate temperature (37 °C) and pH (7.0) for 12 h. Terminate the reaction by boiling for 5 min and then take samples. At the same time, three sets of negative controls were set up to exclude non-specific reactions and other interfering factors: namely, the CAG buffer solution without enzyme, the blank system containing only enzyme and buffer, and the CAG buffer solution containing boiled-inactivated enzyme. After sampling at the set time points, extract three times with equal volume of ethyl acetate. The extract is concentrated by vacuum rotary evaporation and dissolved in 10% acetonitrile. It is subjected to high-performance liquid chromatography for high-performance liquid chromatography analysis.

### 2.10 Enzymatic characteristics of recombinant BgsH

The enzymatic activity of BgsH was determined by continuously measuring its ability to hydrolyze the substrate CAG for 30 min at 37 °C and pH 7.0. The total volume of the reaction system was 150 mL, containing 10 U/mL BgsH and 0.1% (w/v) of the substrate. At the end of the reaction, an equal volume of ethyl acetate was added, and the product was analyzed by high-performance liquid chromatography (HPLC). One unit (1 U) of enzyme activity was defined as the amount of enzyme required to hydrolyze 1 μmol of CAG per minute under the test conditions. All data were the average values of three independent experiments.

In order to study the effects of pH and temperature on the activity of purified BgsH, the reaction system containing 50 mM citrate buffer (pH 7.0) was incubated at different temperatures to determine the optimal temperature, with all other conditions remaining unchanged. To determine the optimal pH value, To determine the optimal pH value, the substrate was reacted with the enzyme in different 50 mM buffers at 37 °C (citrate buffer for pH 3.0–6.0, phosphate potassium buffer for pH 6.0–7.5, Tris HCl buffer for pH 7.5–9.0, Na₂HPO₄-NaOH buffer for pH 10.0–12.0), and then the remaining activity was measured. Thermal stability was evaluated by incubating the enzyme in potassium phosphate buffer (pH 7.0) at different temperatures for 1 h, and then measuring the remaining activity. pH stability was determined by dialyzing the enzyme in different 50 mM buffers at 4 °C for 1 h and measuring the remaining activity. After determining the optimal pH and temperature of the enzyme, under these optimized conditions, the reaction rate at different substrate concentrations was measured.

### 2.11 Optimization of calycosin catalyzed by recombinant BgsH

Meanwhile, the ability of the purified enzyme to produce CA under optimized conditions was studied. Different concentrations (0.05, 0.1, 0.15, and 0.2%) of CA were incubated with the purified enzyme, and the bioconversion efficiency was analyzed by HPLC.

### 2.12 Data statistics and analysis

All experiments were performed in three independent biological replicates. Data are expressed as mean ± standard deviation (SD). Statistical significance was evaluated by one-way ANOVA followed by Tukey’s post-hoc test, with ***p < 0.05 considered significant.”

## 3 Results

### 3.1 Morphological observation and phylogenetic analysis of isolated strains from raw milk

The strain was isolated from raw milk in Mudan District, Heze City, Shandong Province. After 48 h of anaerobic cultivation at 37 °C on an MRS solid plate, the colonies were round, milky white, smooth and moist, with neat edges (Figure 1), and were facultative anaerobic, not capable of liquefying gelatin. The above strain was inoculated into an MRS liquid culture medium for 24 h of cultivation, centrifuged at 8000 rpm for 1 min, and the dry weight of the cells was measured as 4.75 × 10^−3^ ± 0.00065 g/mL. Thus, it can be concluded that strain has a strong reproductive ability.

**Figure 1:**
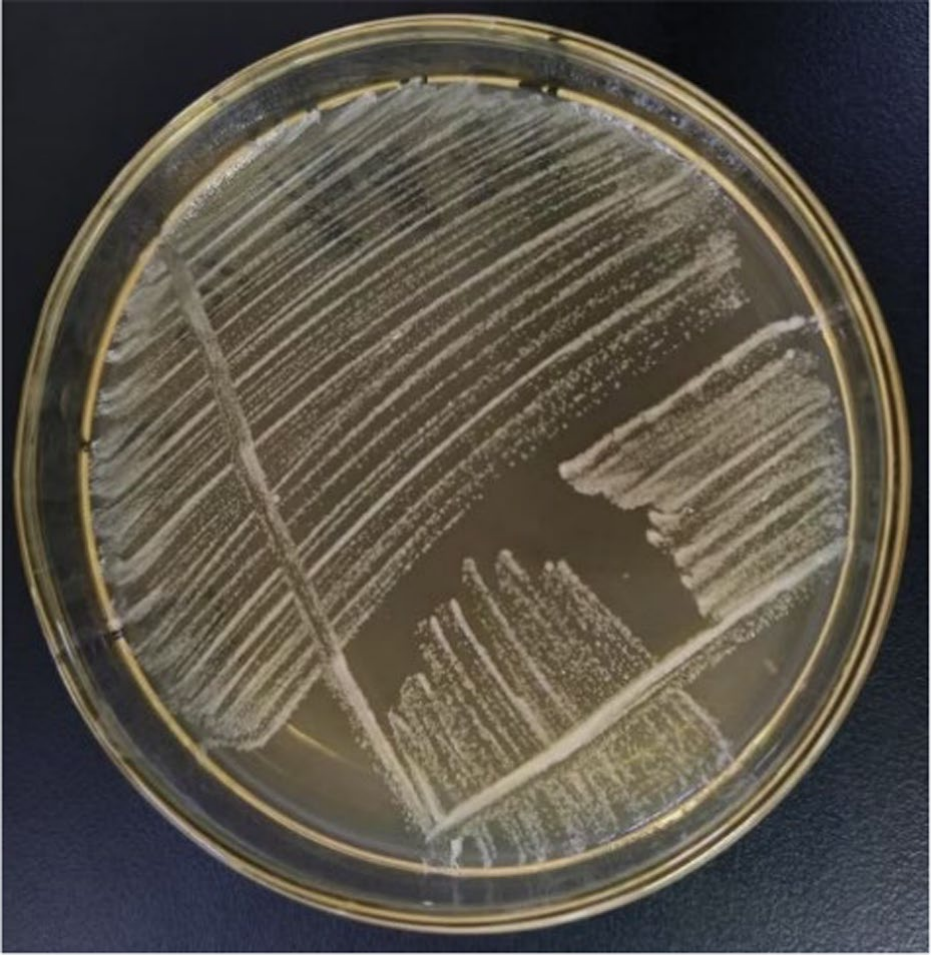
The growth morphology diagram of the strain.

Using the genomic DNA of the strain as the template, PCR amplification was performed using the universal primers for bacterial 16S rRNA to obtain a 1,500 bp fragment of the 16S rRNA gene. The obtained 16S rRNA gene sequence was uploaded to the NCBI database, with the sequence number PZ724680.1.^4^ The phylogenetic tree was constructed using the neighbor-joining method with MEGA software (Figure 2). This strain clustered in the same evolutionary branch with *L. helveticus* NBRC 15019, and had an extremely high bootstrap support rate. Based on the morphological characteristics, physiological and biochemical properties, and the analysis results of the 16S rRNA gene sequence, this strain was identified as *L. helveticus*, and was named HP-B1137. This strain was preserved at the Chinese Type Culture Collection Center (address: Wuhan, Wuhan University), with the preservation number CCTCC M 20261780.

**Figure 2:**
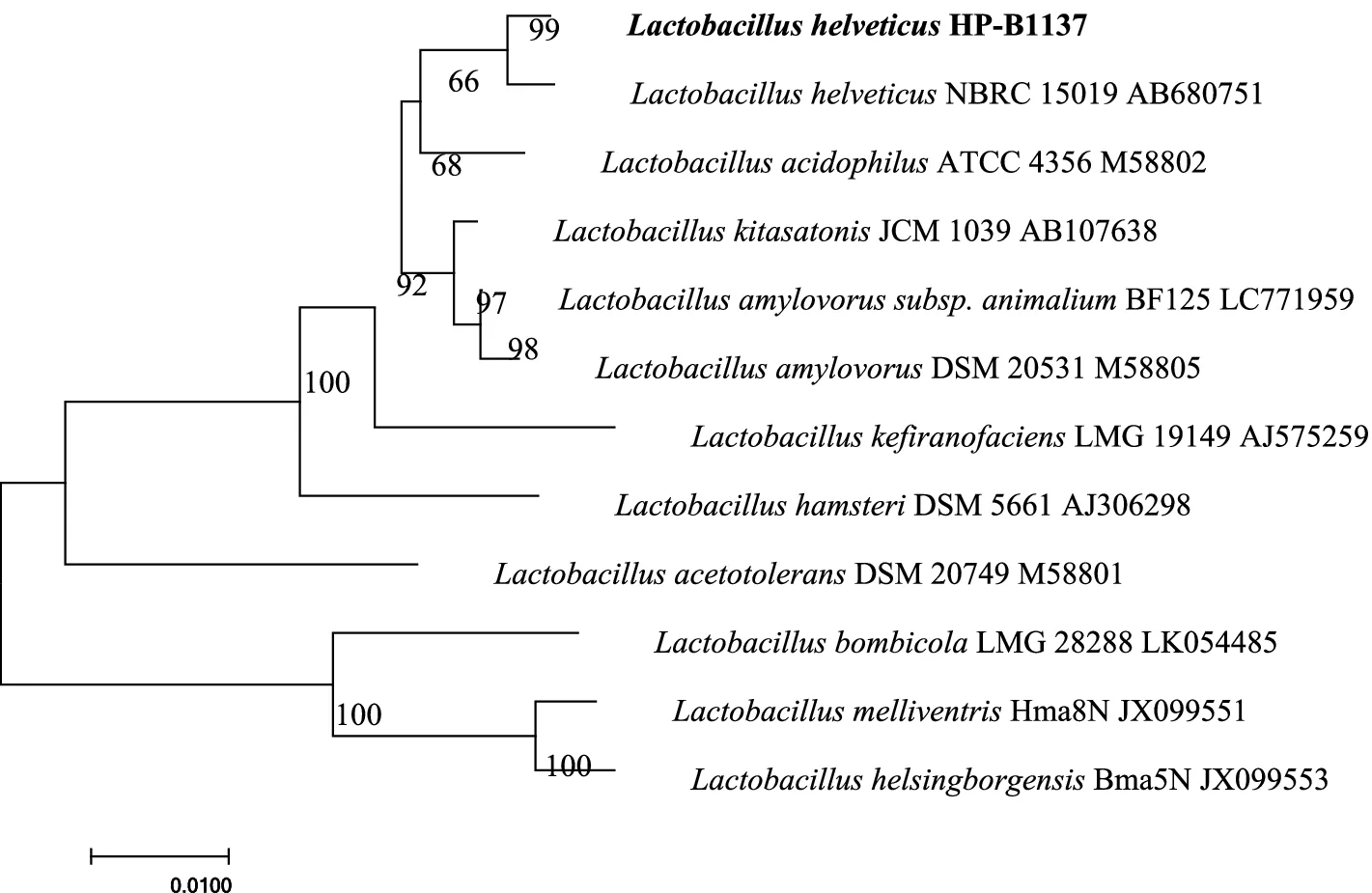
Phylogenetic tree constructed based on 16S rRNA gene sequence by neighbor-joining method, showing the phylogenetic relationship between *L. helveticus* HP-B1137 and closely related species.

### 3.2 Genomic characteristics of *Lactobacillus helveticus* HP-B1137

Based on the hybrid assembly strategy combining Nanopore third-generation sequencing and Illumina second-generation sequencing, the complete genome map of *L. helveticus* HP-B1137 was obtained (Figure 3). The results of genome sequence analysis (Supplementary Table 1) showed that the genome of HP-B1137 consists of one circular chromosome. The chromosome size is 2,098,901 bp, with a GC content of 36.83%. The gene prediction results showed that the genome encodes 2,161 CDs, 63 tRNAs, and 12 rRNAs. The average gene length is 873 bp, and the proportion of coding regions in the total genome length is approximately 87.6%. In addition to the chromosome, two plasmids were identified, with sizes of 23,906 bp and 6,414 bp, and GC contents of 38.54 and 34.49%, respectively (Supplementary Figure 1). The above whole genomic sequence data, plasmid 1 data and plasmid 2 data have been submitted to the NCBI database, with the accession number NZ_JCAULI000000000.1,^5^ NZ_JCAULI010000002.1^6^ and NZ_JCAULI010000003.1.^7^

**Figure 3:**
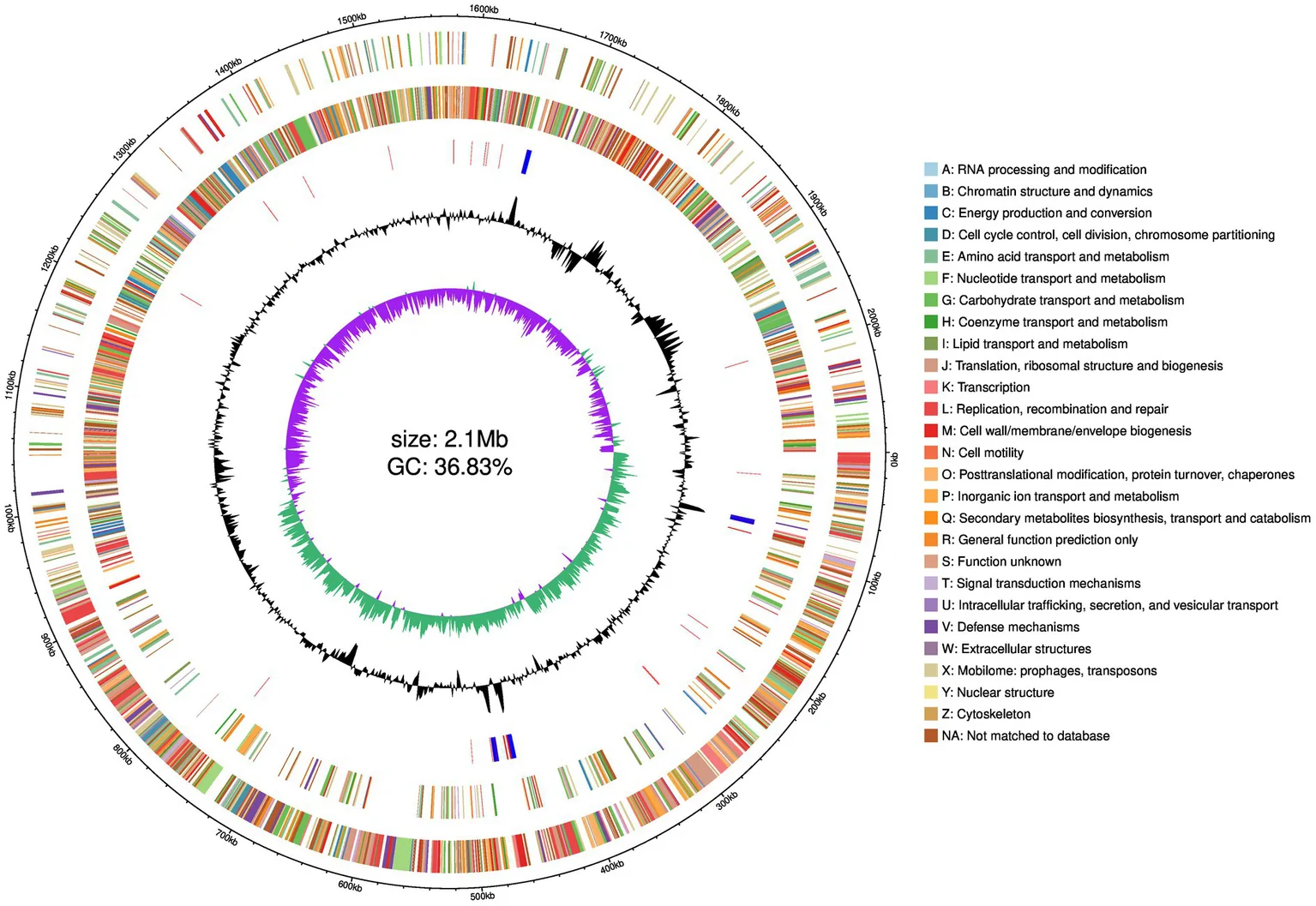
Circular genome map of *L. helveticus* HP-B1137. From outside to inside: the first circle, genome coordinates. The second circle, genes on the positive strand of the genome sequence, with different colors representing different COG functional classifications. The third circle, genes on the negative strand of the genome sequence, with different colors representing different COG functional classifications. The fourth circle, rRNA and tRNA in the genome sequence, rRNA in blue and tRNA in red. The fifth circle, G+C content curve with a 2000 bp sliding window. The sixth circle, G+C skew curve with a 2000 bp sliding window.

### 3.3 Genome annotation and functional analysis of *Lactobacillus helveticus* HP-B1137

The predicted 2,161 protein-coding gene sequences were, respectively, compared with public databases such as COG, KEGG, GO, Pfam, UniProt, RefSeq, NR and TIGRFAMs for functional annotation (Supplementary Table 2). The results showed that all 2,161 genes (100.00%) received at least one type of functional annotation from these databases. The genes annotated to NR and UniProt databases were all 2,161 (100.00%), the genes annotated to the RefSeq database were 2,145 (99.26%), the genes annotated to the COG database were 1,763 (81.58%), the genes annotated to the Pfam database were 1,696 (78.48%), the genes annotated to the GO and KEGG databases were 1,591 (73.62%) and 1,590 (73.58%) respectively, the genes annotated to the Pathway were 760 (35.17%), and the genes annotated to the TIGRFAMs database were 1,064 (49.24%).

The COG functional classification (Supplementary Figure 3) results show that the functional category with the widest distribution of annotated genes is “Translation, Ribosome Structure and Biosynthesis” (Class J), which is annotated to approximately 184 genes; “General Function Prediction Only” (Class R) is annotated to approximately 177 genes; “Mobilome, Prophages Transposons” (Class X) is annotated to approximately 168 genes; “Amino Acid Transport and Metabolism” (Class E) is annotated to approximately 165 genes; “Carbohydrate Transport and Metabolism” (Class G), which is annotated to approximately 157 genes; “Transcription” (Class K) is annotated to approximately 135 genes; “Replication, Recombination and Repair” (Class L) is annotated to approximately 120 genes. These results are highly consistent with the physiological characteristics of *L. helveticus* as a lactic acid bacteria that relies on glycolysis for energy supply and rapid proliferation.

The GO function classification (Supplementary Figure 5) results show that in terms of biological processes, the most enriched entries include amino acid metabolism process, methylation, protein folding and transport, carbohydrate metabolism process, etc.; in terms of molecular functions, the most enriched entries are binding activities (ATP binding, DNA binding, *etc.*) and catalytic activities (ATP hydrolase activity, hydrolase activity, and peptidase activity, etc.); in terms of cellular components, the most enriched entries are reductase complexes, cytosol, and ribosomes.

The KEGG pathway analysis (Supplementary Figure 4) results showed that the annotated genes were mainly enriched in carbohydrate metabolism pathways (accounting for approximately 17.7%), amino acid metabolism pathways (about 11.6%), nucleotide metabolism pathways (about 10.8%), lipid metabolism pathways (about 6.8%), and transmembrane transport (about 13.8%). The annotation results from the CAZy (Carbohydrate-Active Enzymes) database (Supplementary Table 3) indicated that the HP-B1137 genome contained a total of 29 glycoside hydrolase (GHs) genes, 19 glycosyltransferase (GTs) genes, 1 carbohydrate esterase (CEs) gene, 1 auxiliary oxidoreductase (AAs) gene, and 1 carbohydrate binding module (CBMs) gene. Among them, the GHs family was the most abundant category of carbohydrate-active enzymes, including multiple subfamilies with β-glucosidase activity such as GH1, GH3, and GH42, providing a genetic basis for the efficient hydrolysis of glycosidic bonds by the strain. Based on Pfam domain annotations, the genes with each domain annotation were statistically summarized, and the top 20 domains with the most annotations were plotted and displayed. The results are shown in the following Supplementary Figure 2, among them, the hydrolase family has over 20 types. After rigorous comparison with Comprehensive Antibiotic Resistance Database (CARD) and Antibiotic Resistance Genes Database (ARDB), no known acquired antibiotic resistance genes or transferable resistance elements were identified in the genome of HP-B1137. The annotation results from Virulence Factors Database (VFDB) indicated that a total of 412 virulence factors were annotated in the genome. After individual review, all of these were housekeeping genes shared by non-pathogenic *Lactobacilli* (such as adhesins, extracellular polysaccharide synthesis proteins, stress response proteins, etc.), and did not contain specific infectious virulence factors of pathogenic bacteria. The prediction results of signal peptides (SignalP 5.0) showed that there were 153 proteins with signal peptides (Supplementary Table 4); the prediction results of transmembrane helices (TMHMM v2.0) indicated that there were 507 transmembrane proteins (Supplementary Table 5), and 66 secretory proteins (Supplementary Table 5). In this study, target protein sequences were annotated against the NR database using Diamond and the results are shown in Supplementary Figure 6, and the species with the highest proportion among the top 10 were identified as *L. helveticus*, accounting for 86.51%. These results confirmed that the *L. helveticus* HP-B1137 has a high level of biological safety. The summary of shared and unique annotations among different databases is shown in Figure 4.

**Figure 4:**
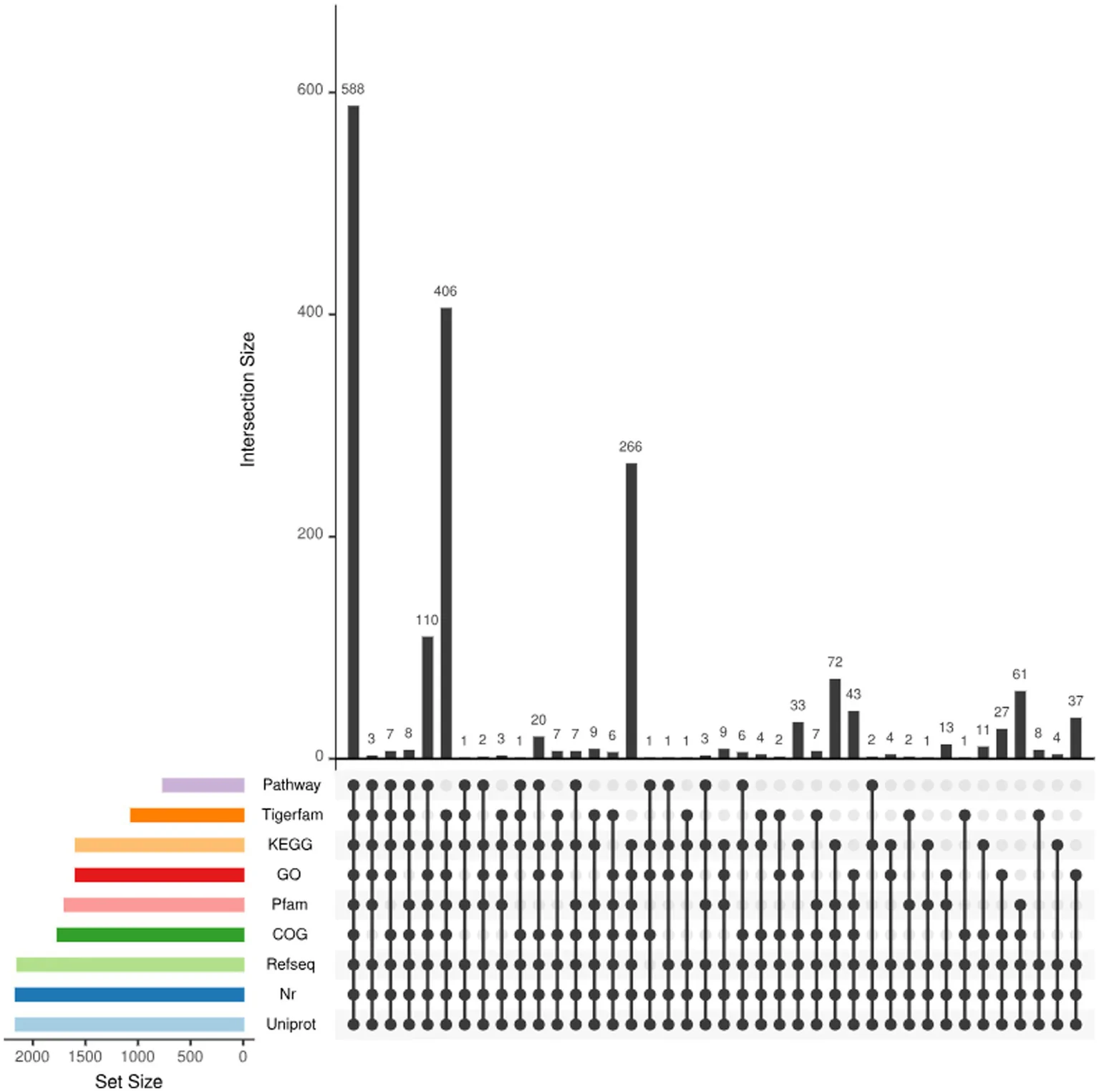
Common and unique gene annotations of *L. helveticus* HP-B1137 in different databases.

### 3.4 Thermal tolerance of *Lactobacillus helveticus* HP-B1137

The *L. helveticus* HP-B1137 bacterial solution was subjected to heat treatment at 37 °C (control), 50 °C and 70 °C for 1, 2 and 3 min respectively, and then the colony counts on the plates were conducted (Figure 5A). The results showed that the viable bacterial counts after 50 °C heat treatment for 1, 2 and 3 min were 8.3 × 10^8^, 1.34 × 10^9^ and 8.2 × 10^8^ CFU/mL respectively, which showed no significant decrease compared to the control group at 37 °C, indicating that the strain has good tolerance to 50 °C high temperature; after 70 °C heat treatment for 1 min, the viable bacterial count was still 3.5 × 10^8^ CFU/mL, while the viable bacterial counts decreased to 0 after 2 min and 3 min of heat treatment. These results indicate that *L. helveticus* HP-B1137 has excellent high-temperature tolerance, providing favorable conditions for its application in spray drying, freeze-drying and heat processing processes of probiotic preparations.

**Figure 5:**
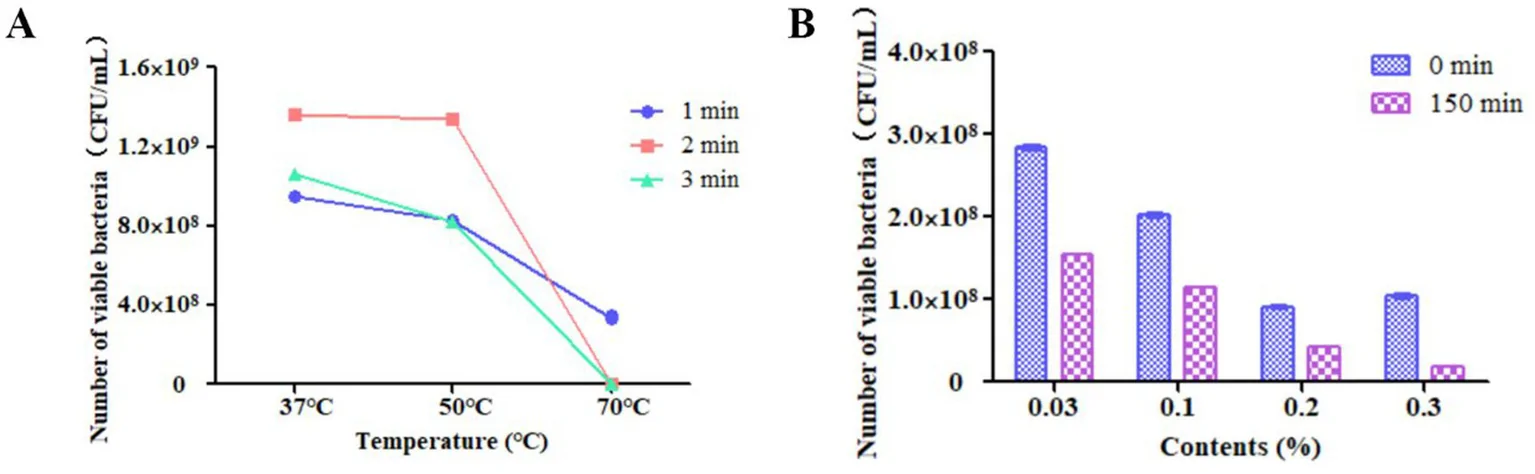
Study on thermal tolerance and bile salt tolerance of *L. helveticus* HP-B1137. **(A)** Thermal tolerance; **(B)** bile salt tolerance.

### 3.5 Acid production and acid tolerance of *Lactobacillus helveticus* HP-B1137

The acid production capacity test results show that when *L. helveticus* HP-B1137 was cultured anaerobically at 37 °C in MRS liquid medium for 12 h, the pH value of the fermentation broth dropped to 4.11; after 24 h of cultivation, the pH further decreased to 4.00; and after 30 h of cultivation, the pH stabilized at 3.99. These results indicate that this strain has a fast acid production rate and strong acid production capacity. It can create an acidic environment in a relatively short time, which is conducive to inhibiting microbial contamination and promoting the balance of intestinal microecology.

The acid resistance test results showed that after being treated in artificial gastric fluid with a pH of 2.7 for 150 min, the viable count of *L. helveticus* HP-B1137 increased from 6.0 × 10^8^ CFU/mL to 6.5 × 10^8^ CFU/mL, with a survival rate of up to 108.33% (Table 1). This result indicates that the strain did not die in a strongly acidic environment but instead showed proliferation. Tatistical analysis by one-way ANOVA revealed no significant difference in viable counts between 0 min and 150 min groups across all tested pH conditions (*p* > 0.05). This may reflect an adaptive stress response rather than genuine proliferation under acidic conditions. It is speculated that the strain induced an adaptive stress response under acid stress. The above results suggest that *L. helveticus* HP-B1137 has extremely strong tolerance to the acidic environment of gastric fluid, ensuring that sufficient viable bacteria reach the intestine and exert probiotic functions.

**Table 1:** Study on acid tolerance of *Lactobacillus helveticus* HP-B1137.

pH	Time (min)	Viable count/(CFU/mL)	Survival rate (%)
2.70	0	6.0 × 10^8^	-
	150	6.5 × 10^8^	108.33%
3.46	0	8.7 × 10^8^	-
	150	9.0 × 10^8^	103.44%
4.75	0	7.7 × 10^8^	-
	150	9.34 × 10^8^	121.30%

### 3.6 Bile salt tolerance of *Lactobacillus helveticus* HP-B1137

After treating *L. helveticus* HP-B1137 in artificial intestinal fluid containing 0.03, 0.1, 0.2, and 0.3% bovine bile salts for 150 min, the survival rate was measured. The results showed that at a bile salt concentration of 0.03%, the survival rate reached 99.65% after 150 min; at a bile salt concentration of 0.1%, the survival rate was 58.82%; at a bile salt concentration of 0.2%, the survival rate was 47.83%; even at a high bile salt concentration of 0.3%, the survival rate still reached 18.87% (Figure 5B). The bile salt concentration in the intestine usually fluctuates within the range of 0.03 to 0.3%. HP-B1137 exhibited good bile salt tolerance within this concentration range and effectively crossed the intestinal bile salt barrier, showing potential for survival in the intestinal environment.

### 3.7 Antibacterial activity of *Lactobacillus helveticus* HP-B1137

The inhibitory activity of the *L. helveticus* HP-B1137 fermentation broth (containing bacteria F and the centrifuged supernatant S) against 11 pathogenic bacteria was determined by the Oxford cup agar diffusion method. The results showed that the *L. helveticus* HP-B1137 fermentation product had a strong inhibitory effect on *Bacillus cereus* ATCC14579, *Klebsiella pneumoniae* ATCC11296, *Acinetobacter baumannii* ATCC19606, *Proteus mirabilis* ATCC29906, drug-resistant *Acinetobacter baumannii* HP-B1160, drug-resistant *Escherichia coli* GTC779, *Salmonella typhi* LT2, *Escherichia coli* O157, drug-resistant *Pseudomonas aeruginosa* JCM5962, drug-resistant *Staphylococcus aureus* DSM20231, and *Shigella* ATCC29903. The diameter of the inhibition zone ranged from 9.83 to 16.37 mm. Moreover, the bacterial suspension without centrifugation had a stronger inhibitory effect compared to the centrifuged supernatant. The inhibitory effect on drug-resistant strains was particularly prominent: among them, the inhibition zone of drug-resistant *Escherichia coli* GTC779 was the largest, with the bacterial fermentation broth (F) reaching 16.37 mm and the supernatant (S) being 15.54 mm. The results determined by the Oxford cup method are shown in the Figure 6. The antibacterial test indicated that the fermentation broth and supernatant of HP-B1137 could effectively inhibit the growth of various pathogenic bacteria.

**Figure 6:**
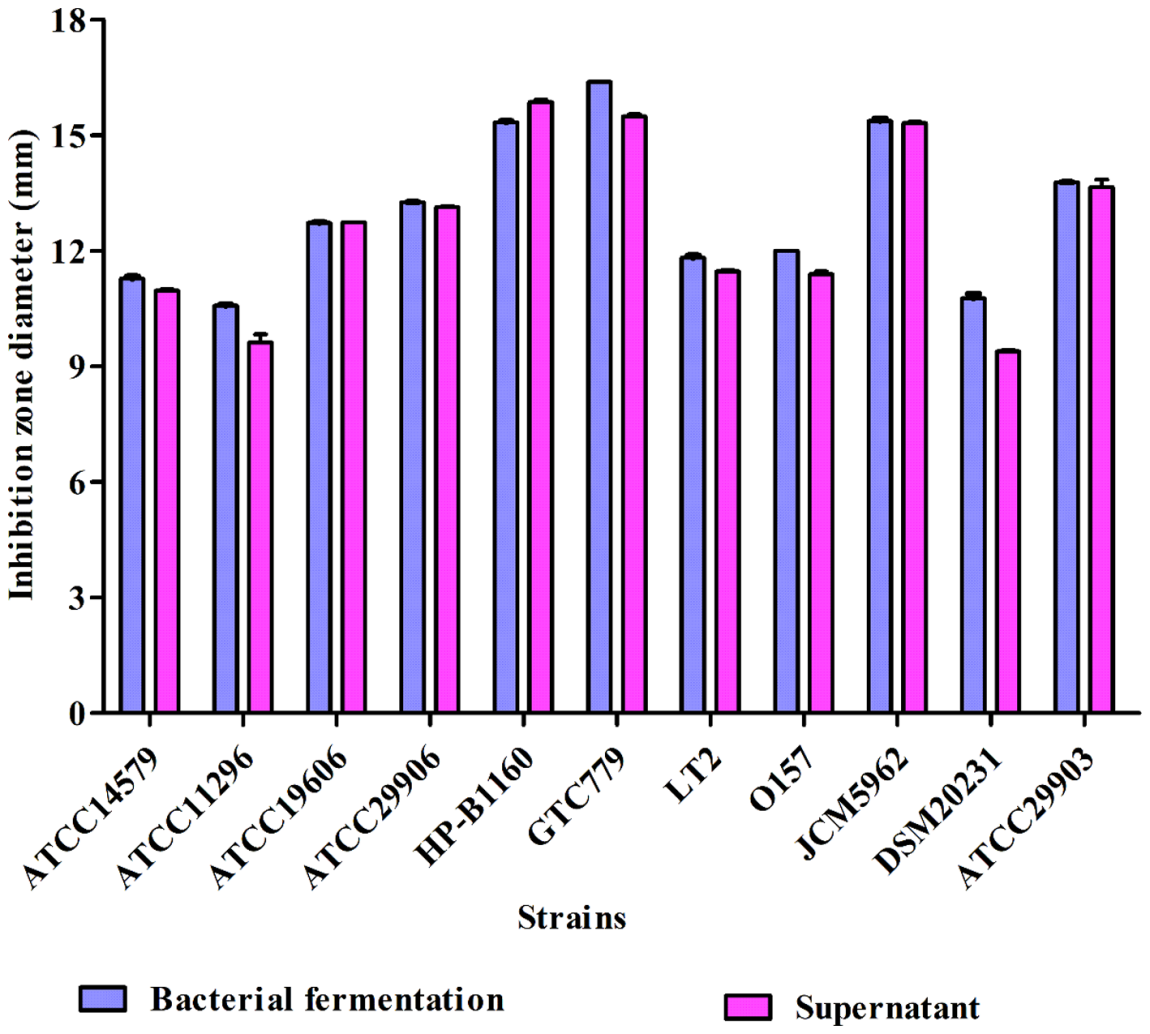
Evaluation of antibacterial effect of *L. helveticus* HP-B137 fermentation broth and supernatant. *Bacillus cereus* ATCC14579, *Klebsiella pneumoniae* ATCC11296, *Acinetobacter baumannii* ATCC19606, *Proteus mirabilis* ATCC29906, drug-resistant *Acinetobacter baumannii* HP-B1160, drug-resistant *Escherichia coli* GTC779, *Salmonella typhi* LT2, *Escherichia coli* O157, *drug-resistant Pseudomonas aeruginosa* JCM5962, drug-resistant *Staphylococcus aureus* DSM20231, and *Shigella* ATCC29903.

### 3.8 Biotransformation of calycosin-β-D-glucoside into calycosin catalyzed by *Lactobacillus helveticus* HP-B1137

Using CAG as the substrate, the dynamic conversion process was monitored by HPLC (with a detection wavelength of 254 nm) using the whole-cell fermentation broth of *L. helveticus* HP-B1137. In the HPLC chromatogram, the retention time of the CAG standard was approximately 12.5 min (Figure 7B1), and that of the CA standard was approximately 19.5 min (Figure 7B4). Taking the content of CAG before fermentation as the indicator, the substrate characteristic peak of CAG almost completely disappeared at 24 h, the conversion rate reached above 90% (Figure 7B2). This result indicates that the whole-cell fermentation system of *L. helveticus* HP-B1137 can efficiently convert CAG into CA.

**Figure 7:**
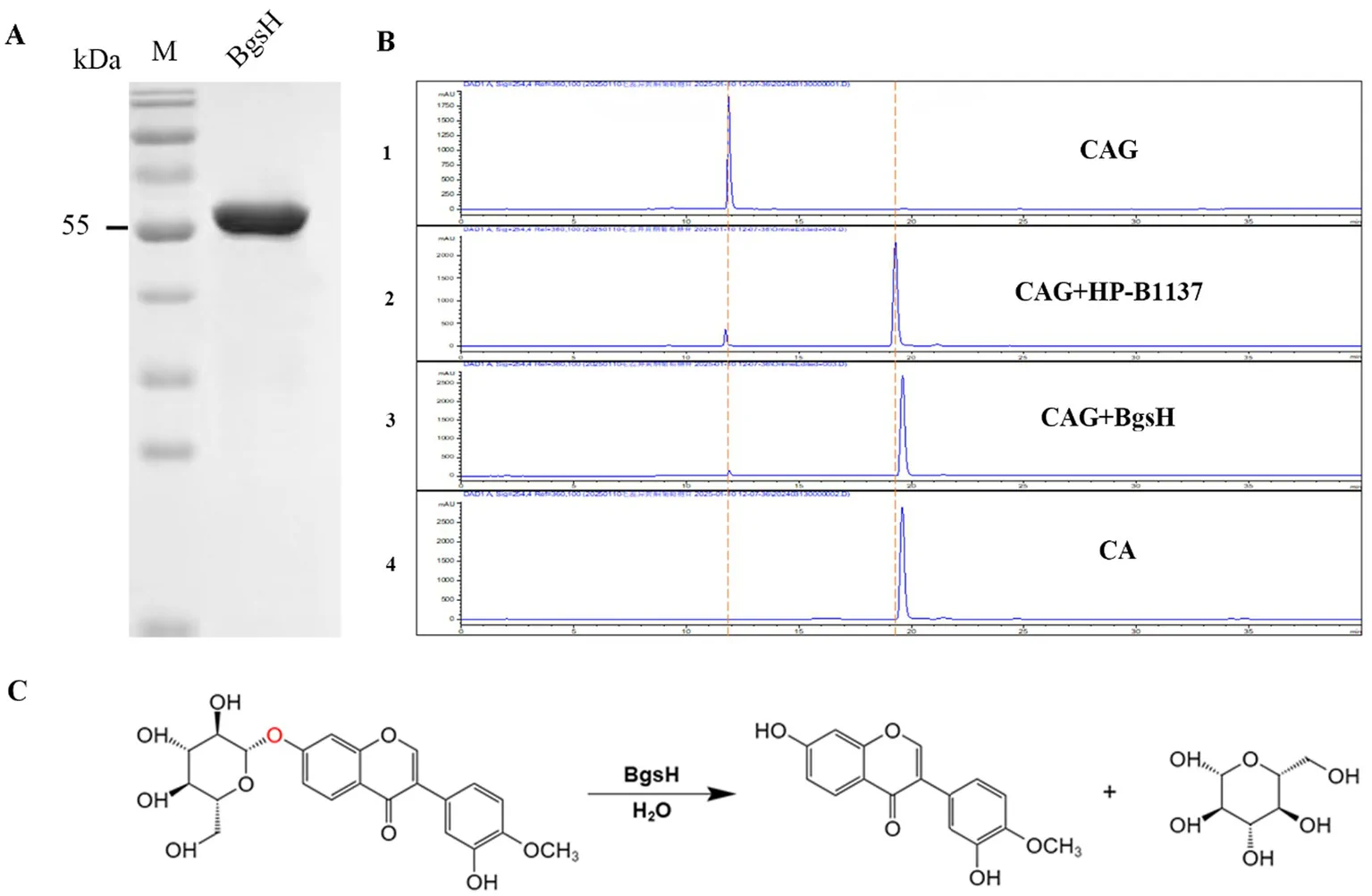
HPLC analysis results of calycosin-β-D-glucoside catalyzed. **(A)** Sodium dodecyl sulfate–polyacrylamide gel electrophoresis (SDS–PAGE) analysis of purified BgsH. Coomassie Brilliant Bluestained12% SDS–polyacrylamide gels are shown. Lane M, molecular weight markers. **(B)** HPLC analysis of the HP-1137 and its enzyme-BgsH. **(C)** The reaction formula of BgsH catalyzing the conversion of CAG to CA. The glucosidic bond attacked by BgsH is highlighted in red.

### 3.9 Heterologous expression and purification of recombinant BgsH

The complete genome annotation results of *L. helveticus* HP-B1137 revealed that a gene encoding 6-phospho-β-D-glucosidase (BgsH) was identified in the genome. This gene is 1,476 bp in length and encodes 492 amino acids, with a theoretical molecular weight of 56.0 kDa. The cloned and expressed recombinant BgsH gene coding sequence of this study has been deposited in the GenBank database, with the accession number PZ730018. After verifying the sequencing results correctly, it was transformed into *Escherichia coli* BL21 (DE3) for heterologous expression. The SDS-PAGE analysis results showed that after induction with IPTG, the recombinant bacteria presented a clear target protein band at approximately 55 kDa (Figure 7A), which was consistent with the theoretical molecular weight. The soluble analysis indicated that the recombinant BgsH protein was mainly present in the supernatant of ultrasonic disruption in an insoluble form. After Ni-NTA affinity chromatography purification, the recombinant BgsH protein was obtained in an electrophoretic pure level, with a single band. The concentration of the purified protein was determined by BCA method and was used for subsequent enzymatic property studies and catalytic conversion experiments. By catalyzing the substrate with the purified recombinant BgsH enzyme, HPLC analysis showed that at 37 °C and pH 7.0 for 12 h, CA products could be detected, complete conversion Figure 7B3. This result further confirmed that BgsH enzyme is the key enzyme responsible for CAG oligoglycosylation conversion. The conversion mechanism of this enzyme is shown in Figure 7C. In the three groups of negative controls set up, no CA was detected, ruling out non-specific reactions and background interference. Collectively, the CAZy classification, the conservation of GH1 catalytic residues, and the unambiguous biochemical evidence establish BgsH as a GH1 β-glucosidase capable of efficiently hydrolyzing the non-phosphorylated isoflavone glucoside CAG, despite its original automated annotation as a 6-phospho-β-glucosidase.

### 3.10 Enzymatic characteristics of recombinant BgsH

Under a pH of 7.0, the activity of the purified recombinant BgsH enzyme was measured at different temperatures (20–50 °C). The results showed that the optimal reaction temperature of BgsH enzyme was 37 °C (Figure 8A). Within the range of 30–40 °C, the enzyme activity remained above 80% of the maximum activity; when the temperature exceeded 50 °C, the enzyme activity significantly decreased. The enzyme activity was measured in different pH buffer systems (pH 3.0–12.0). The results showed that BgsH enzyme had high activity in the pH range of 5.0–8.0, with the optimal reaction pH being 7.0. The enzyme activity significantly decreased when the pH was below 4.0 or above 10 (Figure 8B). The thermal stability test indicated that BgsH enzyme retained more than 85% of the residual activity after incubation at 4 °C and 25 °C for 1 h; after incubation at 50 °C for 1 h, it retained approximately 40% of the activity (Figure 8C), the activity sharply decreased, indicating that this enzyme is a typical mesophilic enzyme and is suitable for catalytic reactions under mild conditions. The pH stability experiment showed that BgsH retained over 85% of its enzymatic activity when stored in a pH buffer solution ranging from 5.0 to 8.0 for 1 h (Figure 8D). If the pH was either higher or lower than this range, the enzymatic activity was lost accordingly.

**Figure 8:**
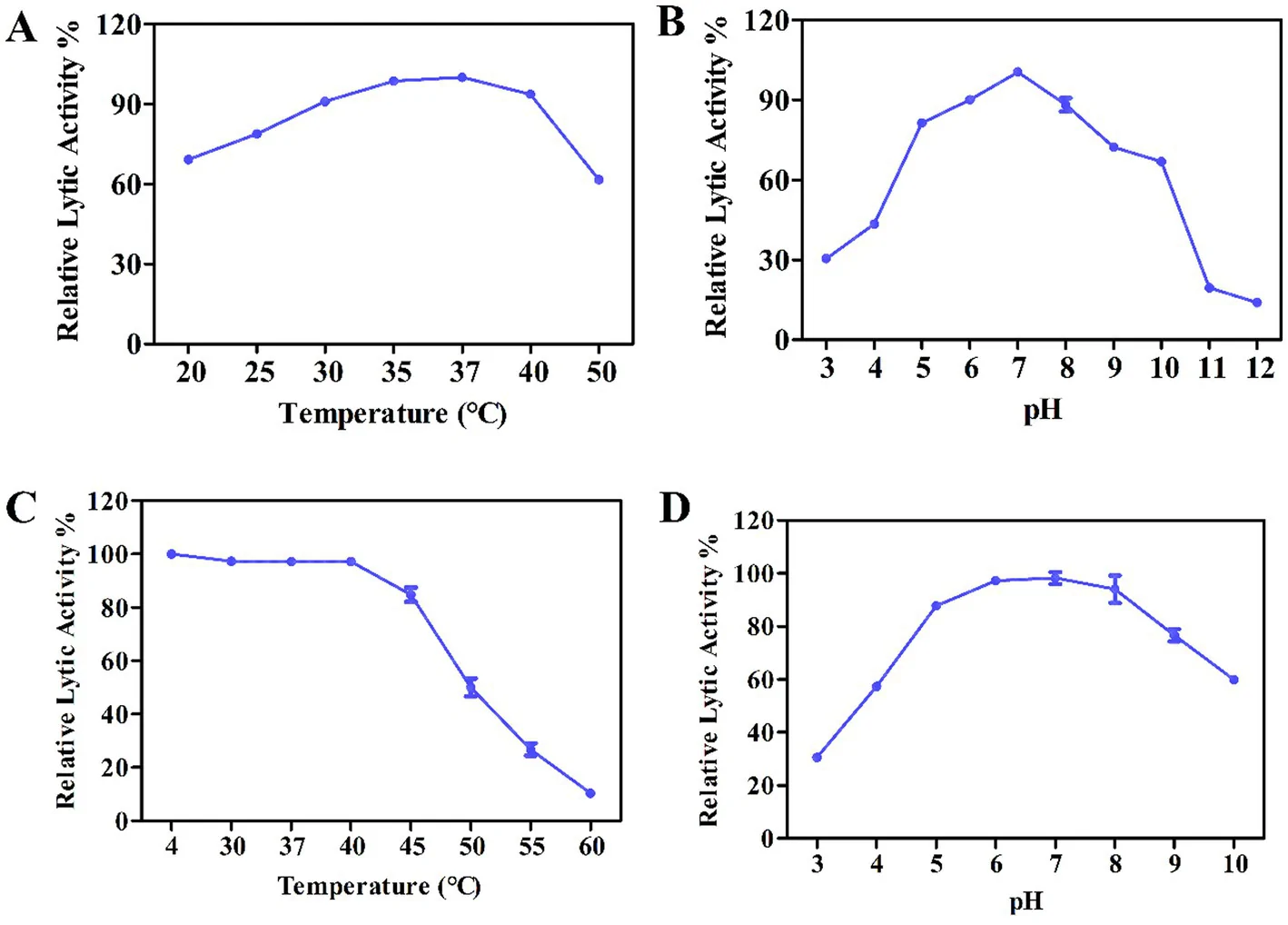
Characterization of the recombinant BgsH. **(A)** Optimal temperature. The value at 37 °C was employed as 100%. **(B)** Optimal pH value. The value at pH 7.0 was employed as 100%. **(C)** Thermal stability. The values were tested by measuring the residual activities after incubating the enzyme at pH 7.0 and various temperatures for 1 h. The value 1 h ago was employed as 100%. **(D)** pH stability. The values were tested by measuring the residual activities after dialysis at 4 °C and various pH values for 1 h. The value 1 h ago was employed as 100%.

### 3.11 Optimization of calycosin biotransformation mediated by purified BgsH

In order to establish the optimal process conditions for the efficient catalysis of CAG to CA by the purified BgsH enzyme, based on the single-factor experiments, systematic optimization was carried out for the substrate concentration and reaction time.

Under the fixed enzyme dosage (10 U/mL) and the optimal reaction conditions (37 °C, pH 7.0), the influence of different substrate concentrations (0.05, 0.1, 0.15, and 0.2%, w/v) on the conversion efficiency was investigated (Figure 9). The HPLC analysis results showed that when the substrate concentration was 0.1% (w/v), the conversion rate could reach over 95% after 12 h of reaction, and complete conversion could be achieved by extending the reaction time to 24 h. When the substrate concentration was increased to 0.15 and 0.2%, the conversion rate decreased under the same reaction time, indicating that high substrate concentration might have a certain inhibitory effect on enzyme activity or product inhibition effect.

**Figure 9:**
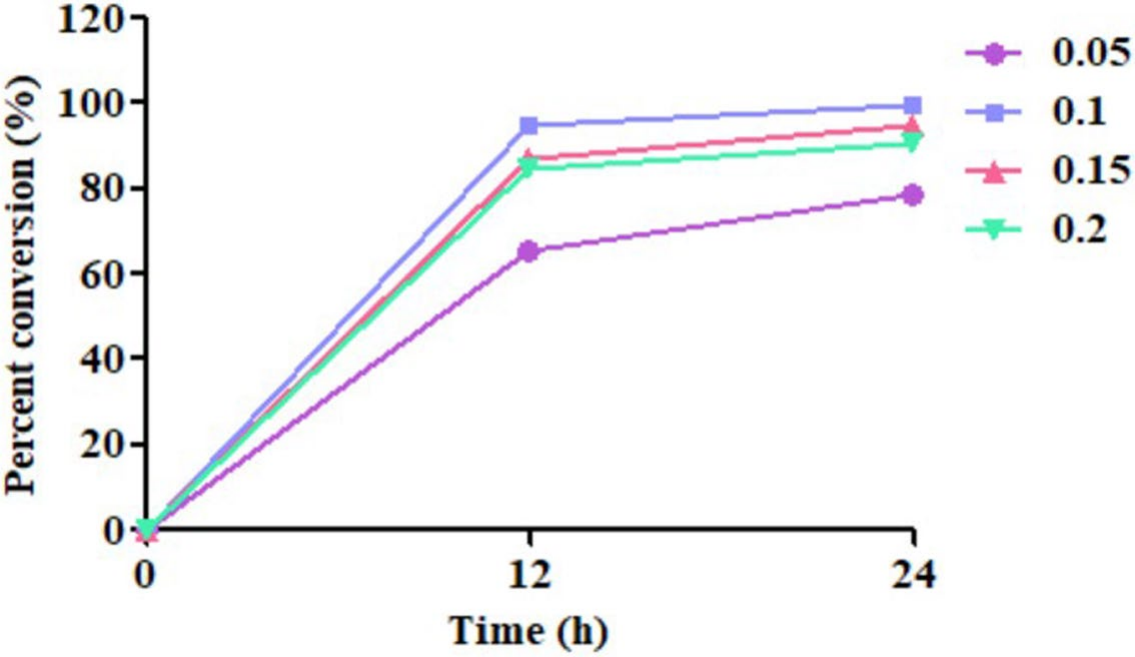
Time course curve of calycosin percent conversion catalyzed by purified BgsH enzyme from *L. helveticus* HP-B1137 under different CAG concentrations. The experiment was performed with three independent replicates.

An amplification verification experiment was conducted under the optimized conditions of 0.1% (w/v) substrate concentration, 10 U/mL enzyme dosage, 37 °C, and pH 7.0. After 12 h of reaction, the yield of CA reached (96.3 ± 2.1)% of the theoretical yield, and after 24 h, it reached (99.8 ± 0.5)% (Figure 9). This achieved a nearly complete conversion of CAG to CA. The above results indicate that the purification of the recombinant BgsH enzyme has an efficient catalytic conversion ability for CAG, providing reliable technical parameters for the enzymatic large-scale preparation of CA.

## 4 Discussion

This study found that the *L. helveticus* HP-B1137 strain can efficiently convert CAG into CA. Based on this finding, the complete genome sequencing and functional annotation of HP-B1137 were conducted, and combined with the genome data, the probiotic properties, environmental tolerance, and antibacterial potential of this strain were systematically verified, and its application value as a functional strain was comprehensively evaluated. At the same time, the enzymatic characteristics of this strain were characterized, the key β-glucosidase (BgsH) involved in the CAG conversion was identified, and its application prospects in the industrial production of CA were further explored. *L. helveticus*, as a recognized safe food-grade microorganism (GRAS), has been included in China’s “List of Microorganisms Suitable for Use in Food,” and the BgsH enzyme produced by it can be used as an effective tool for the safe biological transformation strategy of active aglycon precursors such as anthocyanidin CA. This study cloned, heterologously expressed, and analyzed the enzymatic characteristics of the bgsH gene of *L. helveticus* HP-B1137 to reveal the application efficiency of this unreported biocatalyst in the conversion of CA.

The biological transformation of converting CAG to CA holds significant strategic importance in antioxidant, anti-inflammatory, immune regulation, and cancer adjunctive treatment. (Liu T. et al., 2025). The results of the whole-cell transformation experiment in this study demonstrated that *L. helveticus* HP-B1137 could completely convert 0.5 mg/mL of CAG into CA within 24 h (with a conversion rate of 100%); the purified recombinant BgsH enzyme, under the conditions of 37 °C and pH 7.0, could achieve a conversion rate of over 96.3% for a 0.1% substrate. Compared with the biological transformation efficiency of microbial-derived β-glucosidases reported in existing literature (70–90%), the conversion efficiency of this study has a significant advantage. The high conversion rate may be attributed to the inherent catalytic properties of BgsH enzyme, the appropriate reaction system conditions, and the physicochemical properties of the substrate. As CAG is an isoflavone glycoside, In addition, an appropriate reaction temperature (37 °C) helps to improve the solubility of the substrate, promote the binding of the substrate to the enzyme active center, and thereby enhance the biological transformation efficiency. However, temperature has a dual effect on enzymatic reactions (Ji et al., 2024). While appropriate heating can enhance the solubility of the substrate and the reaction rate, excessively high temperatures can cause the enzyme protein to denature, resulting in a decrease in enzyme stability and limiting the conversion efficiency (Huang et al., 2023). Enzyme activity analysis indicates that the optimal reaction temperature for the BgsH enzyme is 37 °C, and the optimal pH is 7.0; when the temperature exceeds 50 °C or the pH exceeds the range of 5.0 to 8.0, the enzyme activity significantly decreases. Thermal stability tests show that the enzyme retains approximately 40% of its activity after incubation at 50 °C for 1 h. Additionally, an increase in substrate concentration leads to a decrease in the conversion rate and enzyme activity. Existing research has confirmed that the reaction by-product β-D-glucose can act as a competitive inhibitor (Grover and Cushley, 1977; Salgado et al., 2018), feedback inhibiting the activity of glycoside hydrolase and limiting the progress of the enzymatic conversion process. Although high substrate concentration may cause substrate inhibition or product inhibition effects, the characteristic of BgsH enzyme to efficiently convert 0.1% CAG makes it fully suitable for enzymatic large-scale production of CA.

The automated annotation of BgsH as a “6-phospho-β-D-glucosidase” appears inconsistent with its experimentally verified activity toward non-phosphorylated CAG. However, several lines of evidence reconcile this discrepancy. First, CAZy classifies BgsH into GH1, a family functionally diverse but unified by a retaining double-displacement mechanism requiring two catalytic glutamates and no exogenous cofactors (Hill and Reilly, 2008). This contrasts with GH4 6-phospho-β-glucosidases, which strictly depend on NAD^+^ and Mn^2+^ for phosphorylated substrates (Hall et al., 2009). Second, the purified BgsH hydrolyzed non-phosphorylated CAG with >96% conversion (Figure 7), a behavior biochemically consistent with GH1 β-glucosidase activity and incompatible with GH4 specificity. Third, similar annotation–function discrepancies are documented: a recent study identified a GH1 enzyme from *L. plantarum*, initially annotated as a 6-phospho-β-glucosidase, that exhibited clear β-glucosidase activity toward non-phosphorylated substrates (Godse et al., 2025); structural analyses of GH1 enzymes from human microbiome *Lactobacilli* further revealed active-site architectures capable of accommodating both phosphorylated and non-phosphorylated glucosides (Michalska et al., 2013). Thus, the observed hydrolysis of CAG by BgsH is fully compatible with its GH1 classification and highlights the limitation of automated sequence-based function assignment, reinforcing the value of experimental biochemical characterization performed in this study.

Whole-genome sequencing and functional annotation provided a molecular-level explanation for the beneficial and safe characteristics of HP-B1137. No acquired antibiotic resistance genes or transferable drug resistance elements were detected in the genome of this strain, which provided strong genomic evidence for the safety of this strain. The virulence factor annotation results from VFDB were all housekeeping genes shared by non-pathogenic lactic acid bacteria, without the specific infectious virulence factors of pathogenic bacteria. This further confirmed its high safety. The annotation from the CAZy database showed that HP-B1137 carried 29 glycoside hydrolase (GHs) family genes, among which family members such as GH1, GH3, and GH42 usually have extensive β-glucosidase activity, providing a genetic basis for the strain’s efficient hydrolysis of flavonoid glycoside bonds. The mining of these gene resources also provided rich targets for the subsequent development and directed modification of enzyme preparations.

In conclusion, this study thoroughly investigated the genomic characteristics, probiotic properties, and enzymatic properties of *L. helveticus* HP-B1137, confirming that this strain and its BgsH enzyme can efficiently catalyze the conversion of CAG into CA with greater pharmacological activity. This provides high-quality bacterial strain resources and enzymatic tools for the development of functional foods and the green biological manufacturing of active ingredients in traditional Chinese medicine. HP-B1137 breaks through the limitations of traditional β-glucosidase production hosts, which either “only produce enzymes and are not probiotic” or “produce enzymes in low amounts and have complex regulation.” It is a fully cell-based biocatalyst that integrates efficient biological conversion, excellent probiotic properties, extreme environmental tolerance, genomic-level safety, and broad-spectrum antibacterial activity. It has broad application prospects in the development of food and health products using astragalus as the raw material. Subsequent research will focus on the in-depth analysis of the catalytic mechanism of the BgsH, the modification of the enzyme molecule to improve thermal stability and catalytic efficiency, and the verification of the *in vivo* immune regulation and antioxidant efficacy of this strain in animal models.

## Data Availability

The datasets presented in this study can be found in online repositories. The names of the repository/repositories and accession number(s) can be found in the article/Supplementary material.

## Footnotes

1
https://ngdc.cncb.ac.cn/databasecommons/database/id/37

2
https://www.genome.jp/kegg/

3
https://www.geneontology.org/

4
https://www.ncbi.nlm.nih.gov/nuccore/PZ724680.1

5
https://www.ncbi.nlm.nih.gov/nuccore/NZ_JCAULI000000000.1

6
https://www.ncbi.nlm.nih.gov/nuccore/NZ_JCAULI010000002.1

7
https://www.ncbi.nlm.nih.gov/nuccore/NZ_JCAULI010000003.1
