# Supplementary material for: Whole-genome sequencing of Lactobacillus helveticus HP-B1137 and its biotransformation capacity toward calycosin-7-O-β-D-glucoside
Source: Front Microbiol. 2026 Sep 30;17:1962579. doi: 10.3389/fmicb.2026.1962579 (PMC13661924; doi:10.3389/fmicb.2026.1962579)
Supplement: Supplementary file 1 [file Data_Sheet_1.pdf]

Supplementary Material

1 Supplementary Figures and Tables

1.1 Supplementary Tables

Supplementary Table 1. Gene prediction results statistics

Type	Number	Total-len	Average-len	Percentage of genome(%)
Gene	2,271	1,875,677	826	88.09
CDS	2,161	1,847,292	855	86.76
tRNA	63	4,847	77	0.23
23S rRNA	4	11,600	2,900	0.54
16S rRNA	4	6,240	1,560	0.29
5S rRNA	4	444	111	0.02
tmRNA	1	366	366	0.02
misc_rna	34	4,888	144	0.23

Supplementary Table 2. Statistics of coding gene annotation results

Item	Count	Percentage
All	2,161	100.00%
Annotation	2,161	100.00%
KEGG	1,590	73.58%
Pathway	760	35.17%
NR	2,161	100.00%
Uniprot	2,161	100.00%
GO	1,591	73.62%
COG	1,763	81.58%
Pfam	1,696	78.48%
Refseq	2,145	99.26%
Tigerfam	1,064	49.24%

Supplementary Table 3. CAZy commentary among carbohydrate activase database

Class	In a	Description
GHs	29	Glycoside Hydrolases
GTs	19	Glycosy Transferases
PLs	0	Polysaccharide Lyases
CEs	1	Carbohydrate Esterases
AAs	1	Auxiliary Activities
CBMs	1	Carbohydrate-Binding Modules

Note: Class: Carbohydrate Activase Database Functional Classification Name; Num: the number of genes annotated to that classification; Descrition: Description of this classification.

Supplementary Table 4. Signal peptide prediction statistics

Protein Type	Number
Signal peptide protein	153

Supplementary Table 5. Transmembrane protein and secreted protein prediction statistics

Protein Type	Number
Transmembrane protein	507
Secreted protein	66

1.2 Supplementary Figures

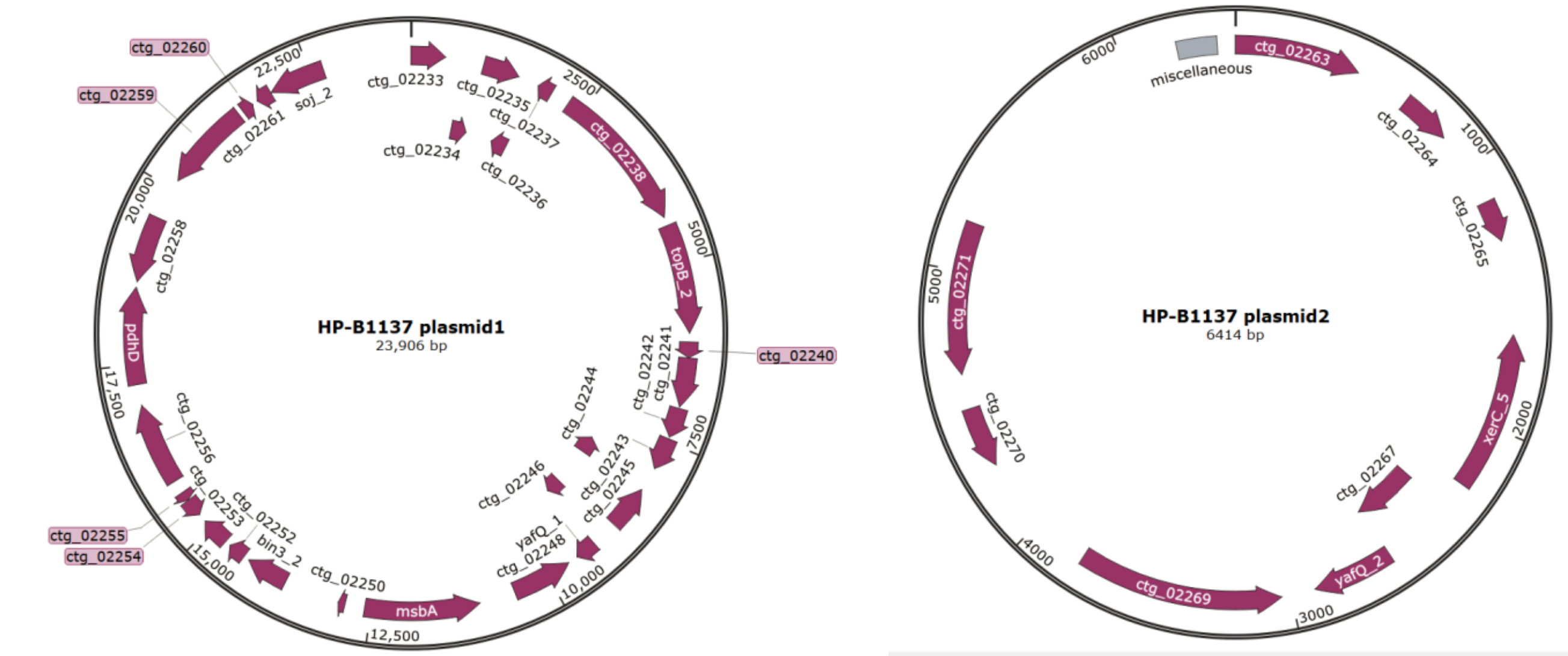

Supplementary Figure 1. The plasmid 1 and plasmid 2 profiles of *L. helveticus* HP-B1137. ..

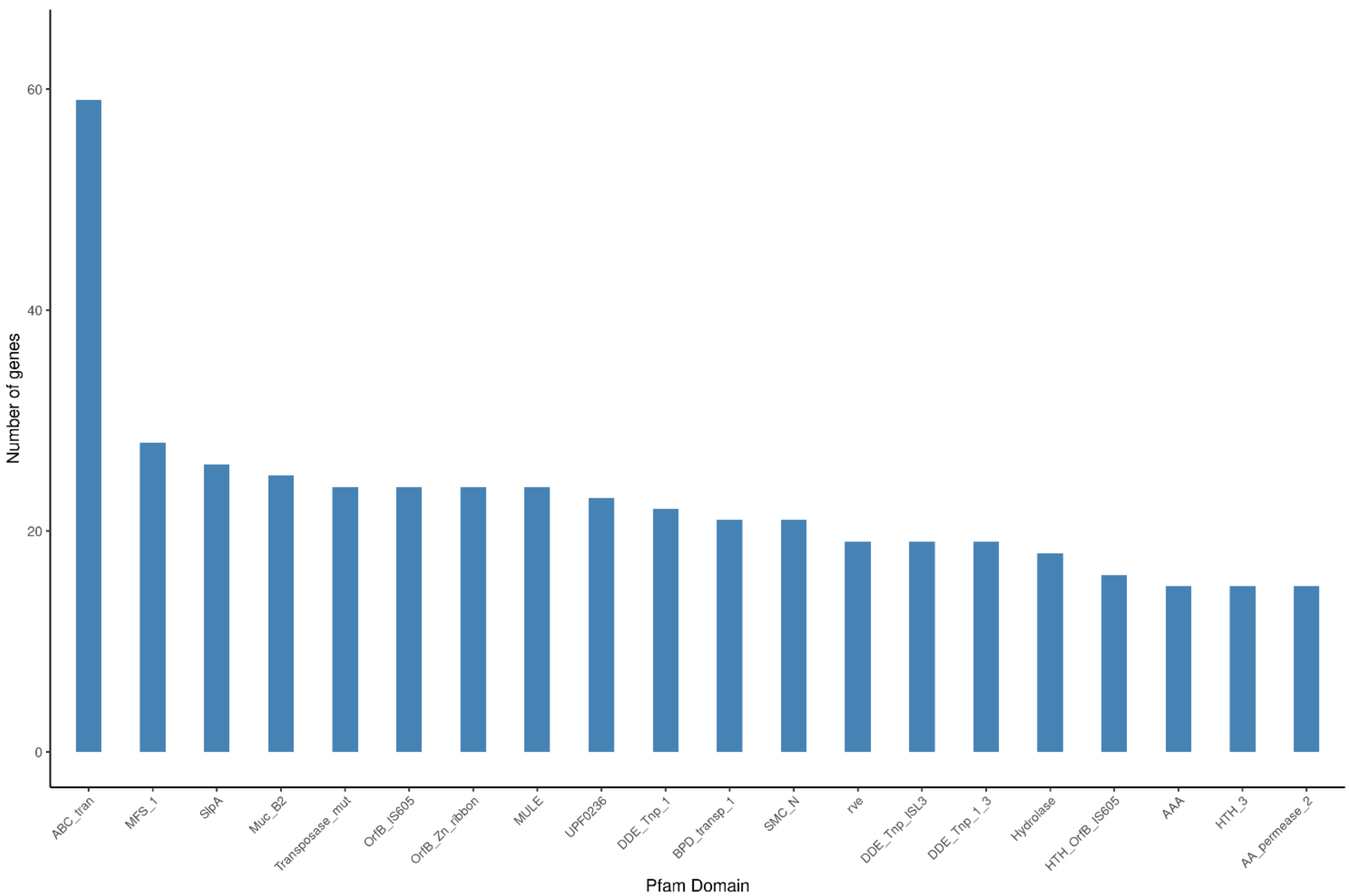

Supplementary Figure 2. The abscissa is the name of the protein family of *L. helveticus* HP-B1137, and the ordinate is the number of genes aligned to the protein family.

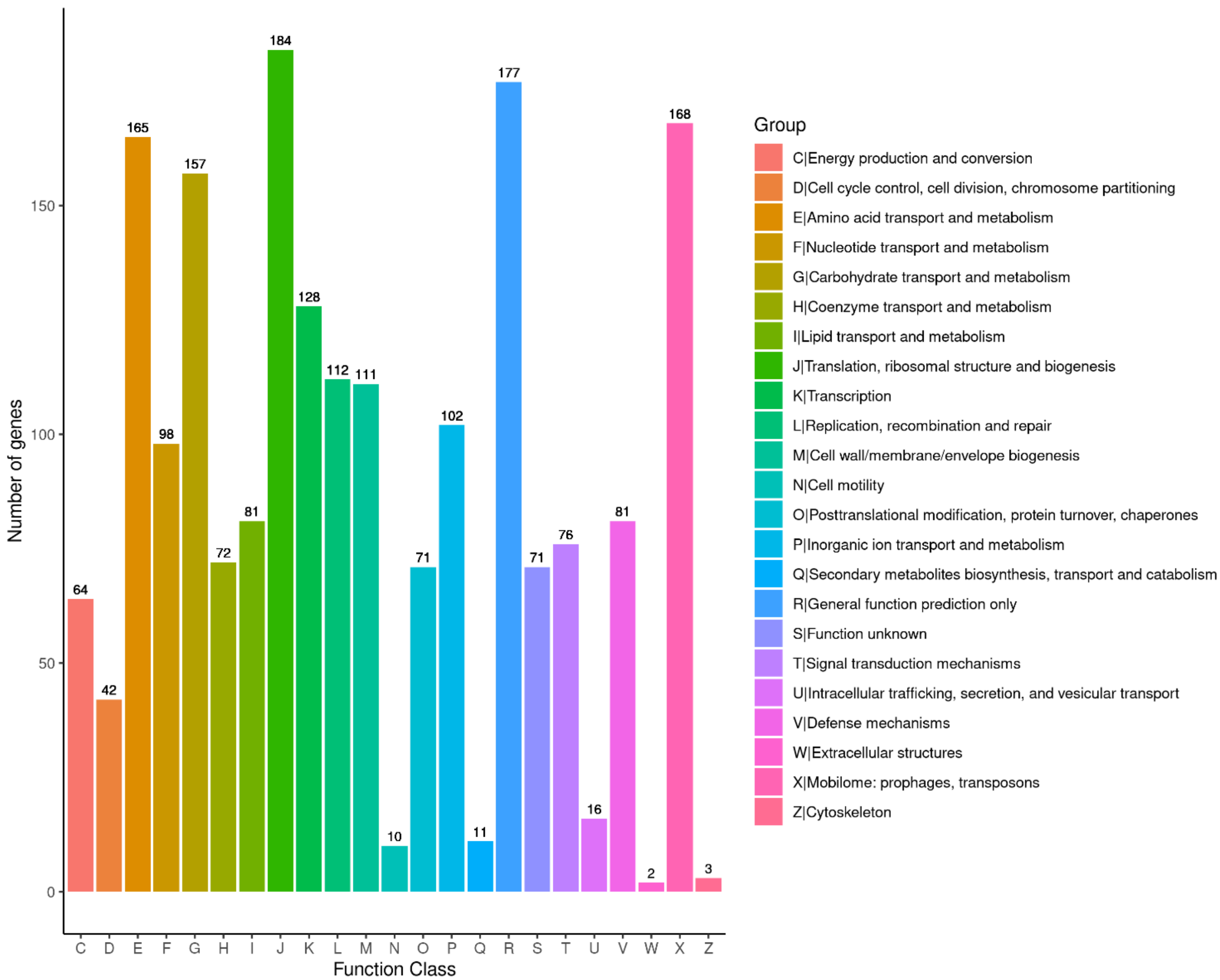

Supplementary Figure 3. The abscissa is the content of each COG classification, and the ordinate is the number of genes. The number of genes reflects the metabolic or physiological tendency in the corresponding period and environment, and scientific explanations can be made by combining the distribution of the research object in each functional category.

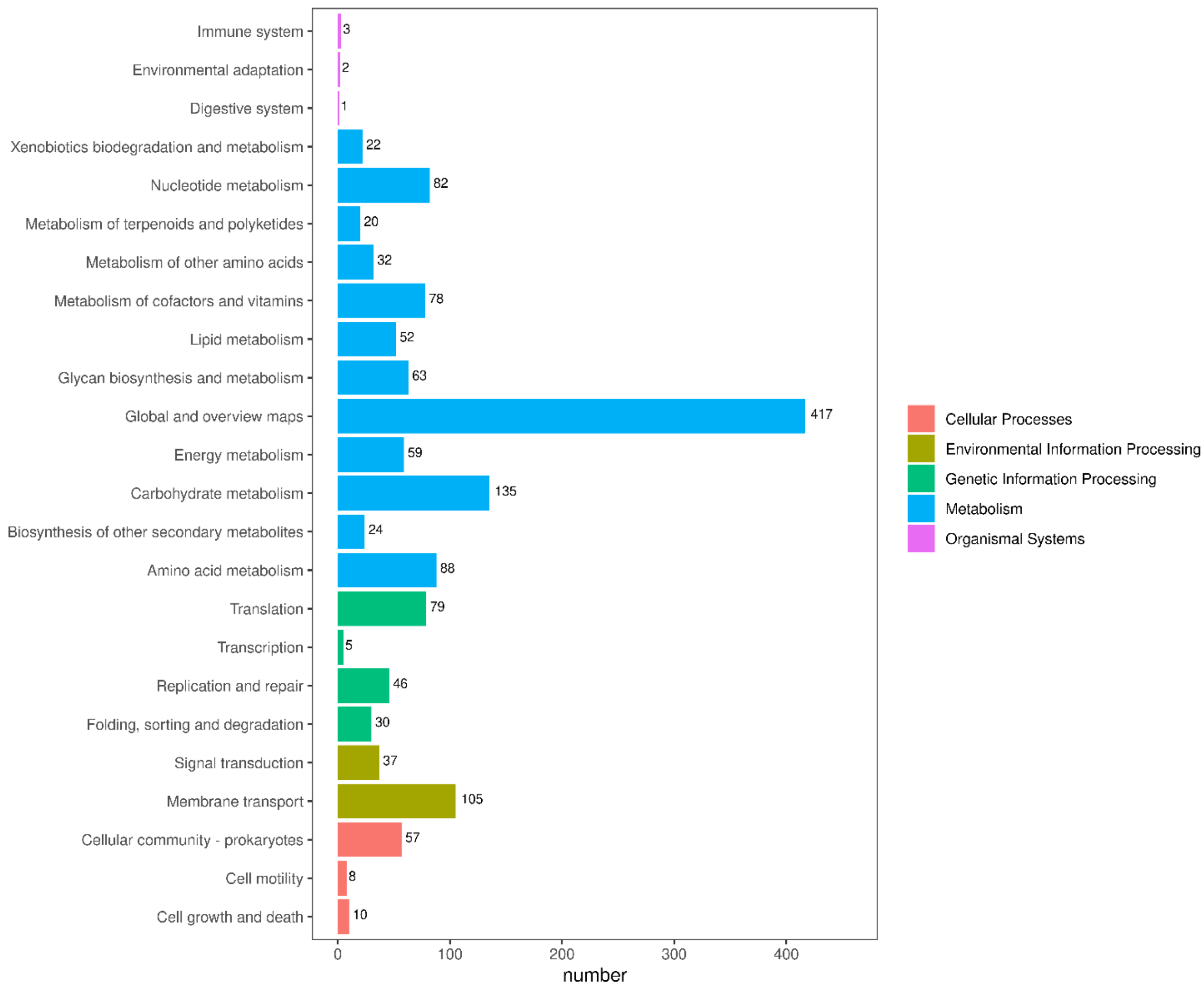

Supplementary Figure 4. The abscissa is the number of genes annotated under Pathway classification, the ordinate is Pathway classification, with different colors representing different major classifications.

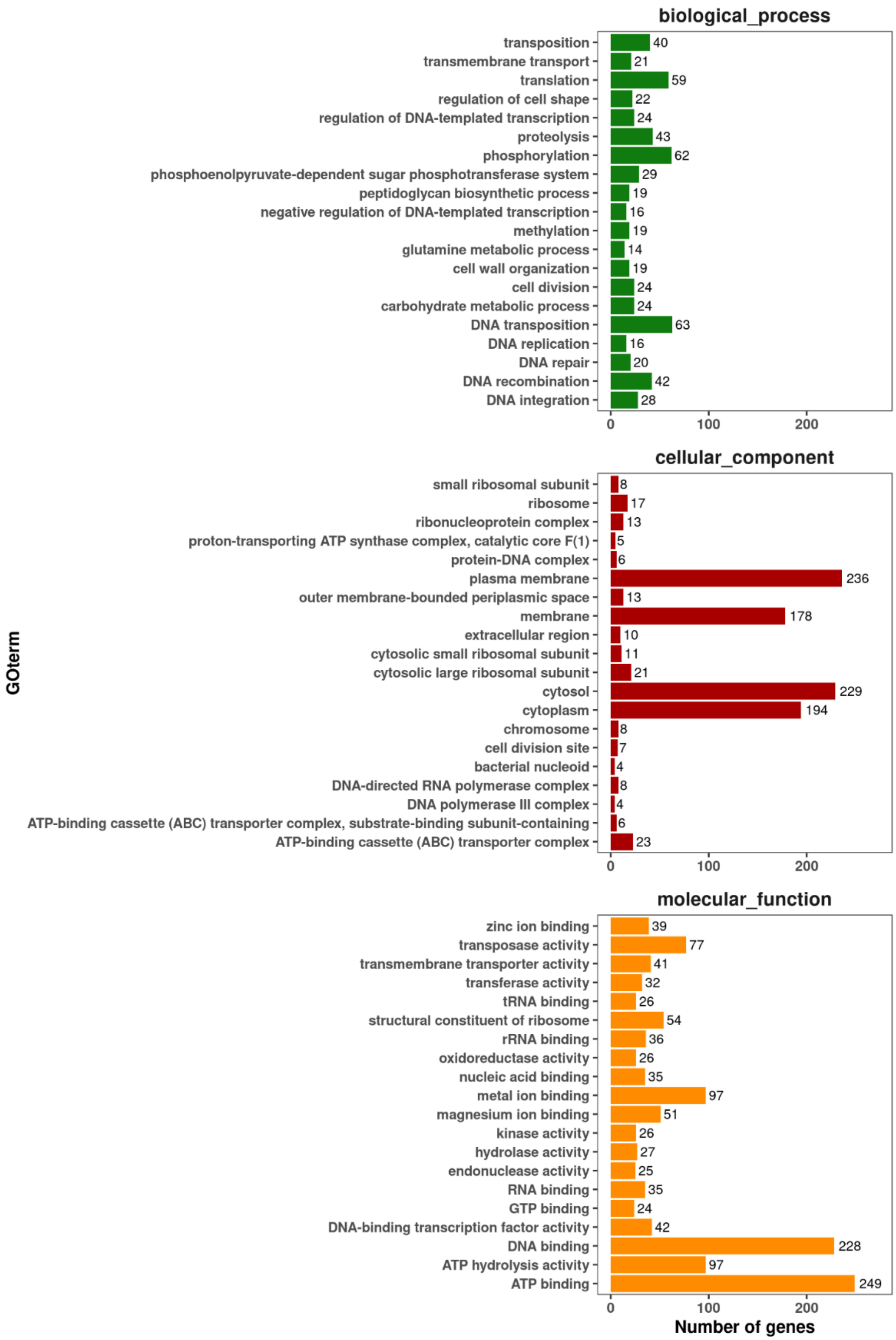

Supplementary Figure 5. The abscissa is the content of each GO classification, and the ordinate is the number of genes.

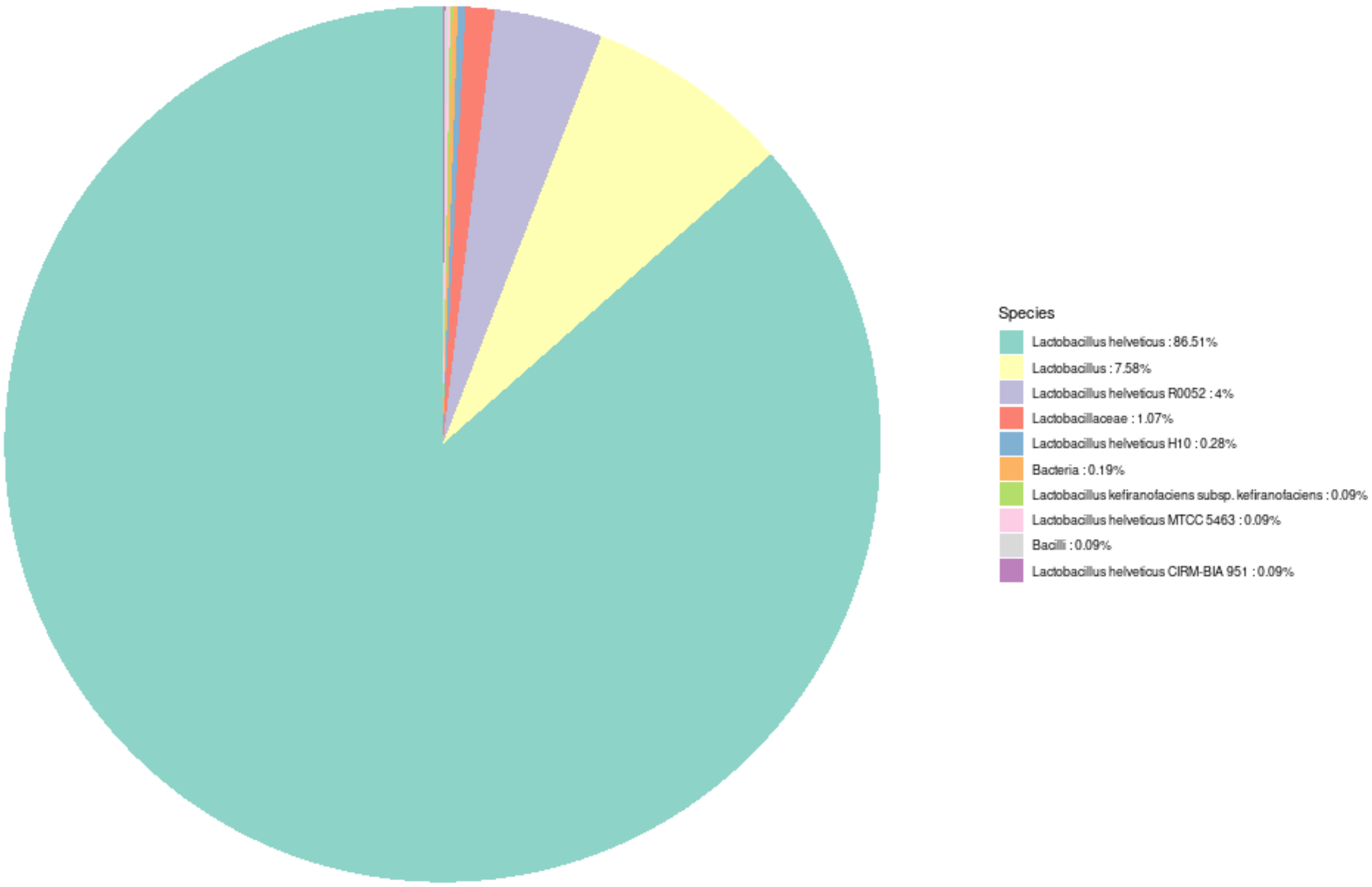

Supplementary Figure 6. NR species distribution map, with different colors representing different species in the figure.
